# Ascending Renal Infection

**Published:** 1904-03-19

**Authors:** 


### ASCENDING RENAL INFECTION.

The manner in which nephritis consecutive to septic conditions of the bladder is produced is a problem which has not hitherto been satisfactorily solved, though some recent experimental work by Dr. John A. Sampson [1] will go a considerable way towards clearing the ground for pathologists and indicating the direction in which future researches may be carried out with a prospect of fruitful discovery.

As Dr. Sampson points out, there are three avenues by which organisms may gain access to the kidneys from the bladder; they are (1) the blood-vessels ; (2) the lymphatics ; and (3) the ureters.

### The Blood-vessels.

Organisms entering the veins of the bladder might reach the kidneys through the general circulation, through the blood-vessels of the ureter, or through anastomotic branches connecting the blood-vessels of the bladder, uterus, ovary, and kidney. That organisms circulating in the blood may pass in fairly large quantities into the urine and so be eliminated from the body, is well known in the case of enteric fever, and this fact led Dr. Sampson to perform some experiments relative to the excretion of circulating micro-organisms by the kidney. In eight dogs he tied the right ureter and injected 2 cc. of a 24-hour bouillon culture of *staphylococcus pyogenes aureus* into the jugular vein. In every instance the kidney whose ureter was tied became the seat of a definite renal infection, while the organism was obtained from both kidneys and also from the bladder. These results would seem to be confirmatory of the view propounded by Cohnheim that micro-organisms circulating in the blood may be excreted by the kidneys, evidences of which process he stated could be seen in the kidneys of guinea-pigs or rabbits which had died of splenic fever. In this connection Dr. Sampson raises a very important question, viz., may the colon bacillus, a very frequent cause of renal infection, and also other organisms, gain access to the circulating blood and be destroyed or excreted by the kidneys without giving rise to any symptoms or in any way injuring the kidney ? Animal experiments suggest that this may take place, for organisms may be injected into the circulating blood and subsequently may be found in the kidneys and also in the blood, and the kidneys appear normal. The presence of micro-organisms in the blood and urine of patients suffering from acute infections also suggests the possibility of the above, for such patients do not necessarily die, and when they do, the kidneys removed afterwards may appear normal.

With regard to the direct connection between the blood-vessels of the bladder and kidneys, Dr. Sampson showed, by injecting the vessels in post-mortem specimens with a coloured solution of gelatine, that there existed anastomotic channels between the vessels of the bladder, ureters and kidneys, and also between those of the bladder and the uterine, ovarian and renal vessels, so that it is conceivable that bacteria might pass from the bladder to the kidney by either of these channels.

### The Lymphatics.

Dr. Sampson carried out some experiments in order to demonstrate, if possible, lymphatic communication between the bladder, ureter and kidney, but the results were unsatisfactory.

### The Ureters.

The main object of his researches was to examine into the possibility or otherwise of the ureters acting as the channels by which infection spread to the kidneys. Theoretically ascending infection might occur by the spread of organisms up the surface of the ureter, or by reflux of urine occurring from the bladder into the ureter. Dr. Sampson showed that the former process may occur if the ureteral orifice be injured, but that something in addition is usually necessary, and this accessory factor is stricture of the ureter, whether due to inflammatory swelling or to other causes ; it is this which interferes with the function of the kidney and causes a stagnation of urine, so that the infected urine may reach a weakened kidney.

Dr. Sampson devoted a great deal of attention to the question of the possibility or otherwise of reflux of urine occurring in health or disease. He points out that anatomically there are three conditions of the bladder to be considered in this connection :—(1) The distended bladder, (2) the contracted bladder, and (3) the flaccid bladder (the normal state of the organ between the acts of micturition). In distension the trigonum is pushed downward, and the course of the ureter becomes more oblique, so that the lumen of the ureter becomes converted into a flattened canal, while the anterior lip of the ureter is forced against the posterior ureteral wall, thus occluding the lumen of the ureter. In contracted bladder the ureter passes through the vesical wall less obliquely, but the lips of the ureteral orifice are brought into much greater prominence, and apparently form an efficient valve. In the normal state the relation between the ureter and the bladder resembles more nearly that of the distended than that of the contracted bladder Passing on to experiments, Dr. Sampson was unable, in the case of 16 dogs, to obtain any reflux from the bladder into the ureters, although in some of the cases renal infection followed the injection of virulent cultures into the bladder; but in these cases stricture of the ureter had been previously caused by operation. It appears, therefore, that so-called ascending renal infection may be caused in dogs without the occurrence of reflux of urine. It has been shown by other observers that reflux of urine may occur in rabbits. As to whether it may occur in human beings is open to question. Dr. Sampson, basing his opinion mainly upon anatomical and physiological considerations, believes that it may take place under pathological conditions which render the ureter rigid, but not under other circumstances; in any case it does not appear to play an important part in the causation of ascending renal infection.

[1] Johns Hopkins Hospital Bulletin.

